# Lead extraction today: a matter of time or a matter of way?

**DOI:** 10.1093/europace/euad325

**Published:** 2023-10-31

**Authors:** Igor Diemberger, Federico Migliore

**Affiliations:** Institute of Cardiology, Department of Medical and Surgical Sciences, University of Bologna, Policlinico S.Orsola-Malpighi, via Massarenti 9, 40138 Bologna, Italy; UOC di Cardiologia, IRCCS Azienda Ospedaliero-Universitaria di Bologna, Dipartimento Cardiotoraco-vascolare, via Massarenti 9, 40138, Bologna, Italy; Department of Cardiac, Thoracic, Vascular Sciences and Public Health, University of Padova, Padua, Italy

**Keywords:** CIEDs, Infection, Endocarditis, Pacemaker, Defibrillator, Complication, Laser, Mechanical transvenous lead extraction

**This editorial refers to ‘Comparison of non-laser and laser transvenous lead extraction: a systematic review and meta-analysis, by Z. Akhtar *et al.,*https://doi.org/10.1093/europace/euad316.**

The evolution of invasive treatments follows similar paths. At first, the focus is the clinical need that emerges as an urgent request for a solution. The initial focus for implantable cardioverter defibrillator (ICD) was post-myocardial infarction (MI) sudden death, the same was the breakthrough of cardiac implantable device (CIED) infections for transvenous lead extraction (TLE) in the early 2000s.^1^ When the first procedure is performed, there is wide enthusiasm and a widespread implementation of the procedure leading to a shift of the focus to the identification of the candidates, barriers to implementation, and definition of the precise technique. For ICD, we saw a shift from post-MI patients to patients with reduced left ventricular ejection fraction, discussions on cost-effective analysis, and the shift from abdominal implant to submuscular and subcutaneous approach. Similarly, it occurred to TLE. While CIED infection was the original indication of TLE, the awareness of a rapid increase in lead malfunction and recalls promoted an increase in non-infective indications to TLE. Although manual traction is an effective technique for recently implanted leads, chronically implanted leads develop strong adherences with surrounding structures requiring additional tools and techniques. This claimed for the development of powered (mechanical and laser) sheaths and different percutaneous approaches including femoral and jugular routes.^1^ However, in specific situations (high-risk procedures, large vegetations, refractory heart failure, valve diseases), ‘hybrid’ or combined approaches may be preferred.^2^

When the procedure is well established, the focus moves to comparison of complications associated with the different approaches such as standard vs. leadless CIED and different devices for TLE.^3^ Safety is an essential clinical endpoint and injury to the superior vena cava (SVC) is the most dangerous and feared complication during TLE.

This is the focus of the paper by Akhtar *et al*.^4^ who reported an interesting meta-analysis on laser-assisted vs. non-laser-assisted approaches for TLE aimed at identifying the safer approach. The result of their huge efforts is an analysis of 68 among 6275 screened papers including 34 laser papers, 30 non-laser papers, and 4 papers including both approaches. After meta-analysing these records, they conclude that non-laser approach resulted to be superior in terms of procedural mortality (pooled rate 0 vs. 0.1%, *P* < 0.01), major complications (pooled rate 0.7 vs. 1.7%, *P* < 0.01), and superior vena cava injury (pooled rate 0 vs. 0.5%, *P* < 0.001) coupled with higher complete TLE success (pooled rate 96.5 vs. 93.8%, *P* < 0.01). In view of the recognized limitations of using data from non-randomized studies, they performed several sensitivity analyses to increase the robustness of their finding confirming their results. The authors also focused on the more recent rotational powered sheaths showing that compared with laser, their use was associated with greater complete success (pooled rate 97.4 vs. 95%, *P* < 0.01) with lower SVC injury (pooled rate 0 vs. 0.7%, *P* < 0.01), but without a significant lower mortality. This is in line with the results by Diaz *et al*.^5^ showing a lower risk of isolated extra-pericardial SVC injury with rotational TLE vs. use of laser sheaths. However, the overall rate of major complication was comparable, suggesting a different nature of major complications using laser or rotational tools with several bias.^6^ Despite the strong efforts by the authors, the results of Akhtar *et al*.^4^ have to be taken with caution: there is a high risk of bias derived from the non-randomized studies included, the possible confounding by indication (since TLE for infection is associated with more overall and procedure-related complications) and the current practice of a multi-step approach to TLE in which the identification of the role of any specific tool to generate the complication seems difficult (*Figure 1A* and *B*). In this regard, a re-assessment of the historical development of TLE approaches highlights a conceptual mistake that could have driven the findings shown by Akhtar *et al*.^4^ The two pros of laser-assisted TLE that were stressed in the PLEXES randomized trial were the superiority over standard mechanical approach (at those time) in terms of complete extraction and procedural time.^7^ The latter, in particular, should not be considered a goal for TLE procedures, especially in case of CIED infections because it is a potential driver of complications especially for less experienced operators. This is in line with the results of the ELECTRA registry in which high-volume centres had less all-cause in-hospital major complications and deaths compared with low-volume ones {2.4% [95% confidence interval (CI) 1.9–3.0%] vs. 4.1% (95% CI 2.7–6.0%), *P* = 0.0146; and 1.2% (95% CI 0.8–1.6%) vs. 2.5% (95% CI 1.5–4.1%) *P* = 0.0088}.^8^ A difference significantly greater than that between laser and non-laser sheaths was reported by Akhtar *et al*. Another finding of this landmark registry by Bongiorni *et al*. is the general low number of complications of TLE which was in contrast with the general consideration that TLE is a high-risk procedure. On a cohort of over 3000 consecutive unselected patients, the primary endpoint of in-hospital procedure-related major complication rate was 1.7% (95% CI 1.3–2.1%) (58/3510 pts) including a mortality of 0.5% (95% CI 0.3–0.8%) (17/3510 pts) and the all-cause in-hospital death occurred in 50 [1.4% (95% CI 1.1–1.9%)] patients.^8^ Notably, while the in-hospital mortality is in line with the findings of Akhtar *et al*.,^4^ the procedure-related mortality is closer to the pooled results of the laser arm (0.46%) rather than the estimated data by the meta-analysis already discussed, or the pooled non-laser data (0.21%). This can raise some doubts on under-reporting bias from some papers included in the meta-analysis despite the efforts of the authors. Besides this, it has to be highlighted that clinical outcomes reported by both papers are comparable to other commonly performed procedures that no physician would consider at the same level of TLE in terms of risks (see *Figure 1C*).^8–11^ However, many authors have recently reported late- and under-referral of patients to TLE centres also for infective indications, including CIED-related endocarditis.^12^ This has a dramatic impact on clinical outcomes of these patients both in terms of survival, overall complications, and costs for the healthcare systems.

**Figure 1:**
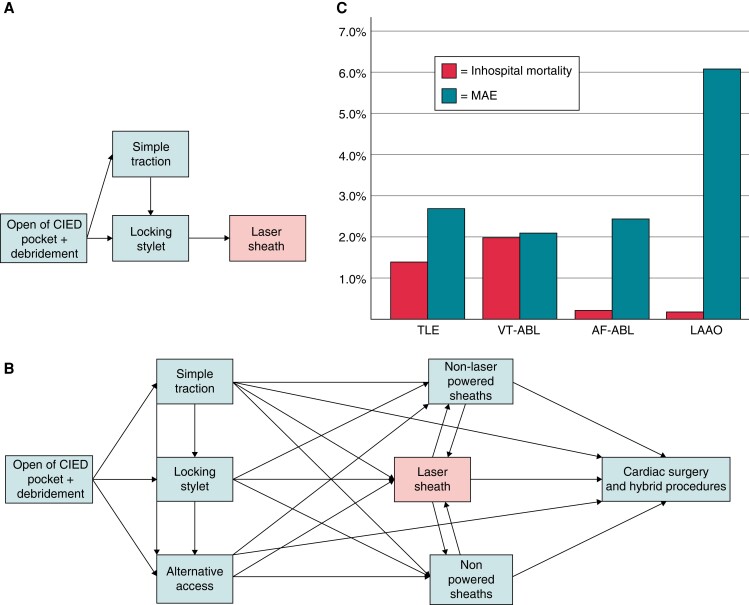
Different approaches to TLE and clinical outcomes compared with other EP procedures. (*A*) Straight laser-focused approach; (*B*) personalized approach including laser sheath; (*C*) comparison of in-hospital outcomes of TLE and other EP procedures according to current clinical practice. Major in-hospital adverse events and mortality for different EP procedures. AF-ABL, atrial fibrillation ablation; LAAO, left atrial appendage occlusion; MAE, major in-hospital adverse events; TLE, transvenous lead extraction; VT-ABL, ventricular tachycardia ablation.

Despite these important considerations, minimization of procedure-related risks has to be pursued as correctly underlined by Akhtar *et al*. through enhancing operators experience to face a procedure that needs careful patient personalization starting before TLE, including proper diagnosis, and following post-TLE management (monitoring, transfer, re-implantation strategy) because non-TLE death is still the leading cause of death for patients with infective indications (which is the more stringent indication to TLE) that have to be managed in a hub-and-spoke perspective.^1,13^ Ongoing research is providing support to this personalization through risk stratification, such as FDG-PET–CT scan,^1^ and identification of risky area.^14^ New technologies have been improved to overcome disasters such as the Bridge Baloon.^1^ Finally, we have not to forget the importance of working in a team. Hybrid procedures involving the collaboration of the TLE operator with the cardiac surgeon can solve tricky cases with the potential risk of a dramatic finale, moving them to a clever minimally invasive approach that can also provide an elegant happy end.^2^

To this regard, The 2018 EHRA Consensus Statement strongly highlights the value of a collaborative and multidisciplinary team approach to properly address the lead extraction management to optimize safety and efficacy.^15^

In conclusion, we thank Akhtar *et al*. in identifying possible area of improvement remembering us that we cannot strictly rely on (rather) new technology to provide effective treatments. On the contrary, during TLE, we have not to embrace a one-fits-all fast approach of doing it, but the best- personalized way of doing it while concentrating our speed to identify and refer our patients in the fastest way. Although new extraction techniques and different tools have been introduced to improve the effectiveness and maintain the safety of TLE, must be emphasized that, one of the long-held tenets of TLE has been the importance and utility of the lead extraction ‘tool box’ implying the having a variety of tools available increases the success and safety of the procedure. Moreover, despite the chosen approach, current outcomes of TLE clearly outweigh the risk of postponing the procedure, especially for patients with CIED infection.

## References

[1] Diemberger I , Boriani G. Infections of Cardiac Implantable Devices: A Comprehensive Guide. Switzerland: Springer Nature; 2020.

[2] Migliore F , Tarzia V, Dall'Aglio PB, Falzone PV, De Lazzari M, Bottio T et al The valuable interaction among cardiac surgeon and electrophysiologist for transvenous rotational mechanical lead extraction. Pacing Clin Electrophysiol 2022;45:92–102. 34699079 10.1111/pace.14396

[3] Boveda S , Marijon E, Lenarczyk R, Iliodromitis KE, Marin F, Defaye P et al Factors influencing the use of leadless or transvenous pacemakers: results of the European Heart Rhythm Association Prospective Survey. Europace 2020;22:667–73. 31960027 10.1093/europace/euz357

[4] Akhtar Z , Kontogiannis C, Georgiopoulos G, Starck C, Leung L WM, Lee S et al Comparison of non-laser and laser transvenous lead extraction: a systematic review and meta-analysis. Europace 2023:euad316. 37882609 10.1093/europace/euad316 PMC10638006

[5] Diaz CL , Guo X, Whitman IR, Marcus GM, Pellegrini CN, Beygui RE et al Reported mortality with rotating sheaths vs. laser sheaths for transvenous lead extraction. Europace 2019;21:1703–9. 31545350 10.1093/europace/euz238

[6] Defaye P , Diemberger I, Rinaldi CA, Hakmi S, Nof E. Mortality during transvenous lead extraction: is there a difference between laser sheaths and rotating sheaths? Europace 2020;22:989. 10.1093/europace/euaa032 32087009

[7] Diemberger I , Mazzotti A, Giulia MB, Cristian M, Matteo M, Letizia ZM et al From lead management to implanted patient management: systematic review and meta-analysis of the last 15 years of experience in lead extraction. Expert Rev Med Devices 2013;10:551–73. 23895081 10.1586/17434440.2013.811837

[8] Bongiorni MG , Kennergren C, Butter C, Deharo JC, Kutarski A, Rinaldi CA et al The European Lead Extraction ConTRolled (ELECTRa) study: a European Heart Rhythm Association (EHRA) registry of transvenous lead extraction outcomes. Eur Heart J 2017;38:2995–3005. 28369414 10.1093/eurheartj/ehx080

[9] Konig S , Ueberham L, Muller-Rothing R, Wiedemann M, Ulbrich M, Sause A et al Catheter ablation of ventricular arrhythmias and in-hospital mortality: insights from the German-wide Helios hospital network of 5052 cases. Europace 2020;22:100–8. 31638643 10.1093/europace/euz260

[10] Munir MB , Khan MZ, Darden D, Abideen Asad ZU, Osman M, Singh GD et al Association of heart failure with procedural complications and in-hospital outcomes from left atrial appendage occlusion device implantation in patients with atrial fibrillation: insights from the national inpatient sample of 62 980 procedures. Europace 2022;24:1451–9. 35613020 10.1093/europace/euac043

[11] Benali K , Khairy P, Hammache N, Petzl A, Da Costa A, Verma A et al Procedure-related complications of catheter ablation for atrial fibrillation. J Am Coll Cardiol 2023;81:2089–99. 37225362 10.1016/j.jacc.2023.03.418

[12] Sciria CT , Kogan EV, Mandler AG, Yeo I, Simon MS, Kim LK et al Low utilization of lead extraction among patients with infective endocarditis and implanted cardiac electronic devices. J Am Coll Cardiol 2023;81:1714–25. 37100488 10.1016/j.jacc.2023.02.042

[13] Diemberger I , Migliore F, Biffi M, Cipriani A, Bertaglia E, Lorenzetti S et al The “subtle” connection between development of cardiac implantable electrical device infection and survival after complete system removal: an observational prospective multicenter study. Int J Cardiol 2018;250:146–9. 29032885 10.1016/j.ijcard.2017.07.061

[14] Patel D , Vatterott P, Piccini J, Epstein LM, Hakmi S, Syed I et al Prospective evaluation of the correlation between gated cardiac computed tomography detected vascular fibrosis and ease of transvenous lead extraction. Circ Arrhythm Electrophysiol 2022;15:e010779. 36306341 10.1161/CIRCEP.121.010779 PMC10503543

[15] Bongiorni MG , Burri H, Deharo JC, Starck C, Kennergren C, Saghy L et al 2018 EHRA expert consensus statement on lead extraction: recommendations on definitions, endpoints, research trial design, and data collection requirements for clinical scientific studies and registries: endorsed by APHRS/HRS/LAHRS. Europace 2018;20:1217. 29566158 10.1093/europace/euy050

